# Advancing medical question answering with a knowledge embedding transformer

**DOI:** 10.1371/journal.pone.0329606

**Published:** 2025-08-18

**Authors:** Xiang Zhu, Mustaqeem Khan, Abdelmalik Taleb-Ahmed, Alice Othmani

**Affiliations:** 1 Laboratoire Images, Signaux et Systémes Intelligents (LiSSi), Université Paris Est Créteil (UPEC), Paris, France; 2 College of Information Technology, United Arab Emirates University (UAEU), Abu Dhabi, United Arab Emirates; 3 Laboratory of IEMN, Centrale Lille, Univ. Polytechnique Hauts-de-France, Valenciennes, France; A’Sharqiyah University, OMAN

## Abstract

Efficient medical question answering is essential for better patient care. Despite progress since Eliza (1966), even advanced LLMs (e.g., GPT-4) struggle with medical data. This study presents a system combining knowledge embedding and transformers. It includes a knowledge understanding layer and an answer generation layer. Tested on the MedQA dataset, it achieved 82.92% accuracy, outperforming GPT-4’s 71.07%. The results demonstrate the system’s ability to deliver accurate and ethical answers. This integrated method improves response speed and quality. Future work will enhance precision, support patient interaction, and integrate multimodal data for improved healthcare query processing.

## Introduction

Healthcare presents both a tremendous opportunity and a significant challenge in developing automated systems, which are proving effective in hospitals, clinics, pharmacies, and home care settings [1]. These systems improve communication between healthcare providers and patients, improving disease management and treatment efficiency. With healthcare chatbots, patients can receive answers to their questions, receive specialized assistance, and autonomously receive healthcare management solutions, improving efficiency and patient care. Over the decades, we have seen significant technological advances since the introduction of Eliza, the pioneering psychotherapist chatbot that used pattern matching in 1966 [2]. These innovations have revolutionized human-computer interactions through chatbot technology.

However, modern systems employ sophisticated techniques, such as pattern recognition, knowledge graphs, and retrieval, along with combinations of transformer-based generative models, to enhance intelligent data processing and decision-making [3]. Despite the advent of powerful tools, such as GPT and large language models, medical datasets often remain challenging due to their complexity and specificity [4, 5]. The system requires extensive medical knowledge, patient-specific data, and sophisticated reasoning capabilities to generate accurate and contextually relevant answers within the medical domain. Medical solutions often fail to achieve high accuracy, flexibility, ease of implementation, and low computational demands simultaneously [6]. The complexity and variability of medical data effectively exacerbate these limitations, providing a balanced solution that improves performance in all these critical dimensions [7, 8].

To address these challenges, we developed a novel question-answering system, the Medical Answering Model (MAM), specifically tailored for the healthcare domain. Our system leverages advanced machine learning algorithms to provide more accurate and reliable responses, enhancing interactions between healthcare providers and patients. This technique may revolutionize patient care by streamlining communication, reducing errors, and facilitating more efficient and personalized treatment. Our approach underscores the importance of integrating advanced technologies with domain-specific expertise to overcome existing limitations and drive meaningful improvements in healthcare.

This research introduces advanced data-processing and optimization techniques to enhance system performance and reasoning abilities. Our contributions are summarized as follows:

- We propose a novel knowledge-embedding transformer that combines knowledge vector similarity computation with self-attention mechanisms to address complex medical questions using a comprehensive medical knowledge base.
- During both fine-tuning and knowledge distillation, we incorporate knowledge embedding techniques to enhance the model’s medical reasoning capabilities.
- To support accurate and context-rich responses, we utilize an extensive medical knowledge base comprising diverse documents. Furthermore, we employ three complementary retrieval strategies to improve information access and retrieval efficiency, thus strengthening the overall performance of the question-answer system in medical domains.

We structure the content of this paper in the following way: the Related Work Section illustrates the related work, and the Proposed Approach Section outlines our proposed solution, describing the integration of the components of the question-answer system. The Experimental and Results Section presents experimental results, showcasing the efficacy of our approach, and the Discussion Section discusses limitations and provides a detailed analysis. Finally, the Conclusions and Future Work section summarizes the key findings and outlines future research directions.

## Related work

Recent advances leverage a combination of cutting-edge technologies. These include pattern recognition, which enables systems to identify and analyze complex data patterns, and knowledge graphs that structure information to improve context understanding [7]. Furthermore, retrieval techniques facilitate efficient access to relevant data, while transformer-based generative models enhance the system’s ability to generate coherent and contextually relevant responses [9, 10]. Together, these technologies improve the accuracy, efficiency, and adaptability of modern intelligent systems, paving the way for more sophisticated applications in various domains [11–13]. Recent studies have introduced transformer-based methods that incorporate retrieval and knowledge graphs [14], demonstrating improved performance and contextual understanding in medical question answering, thereby enhancing the accuracy and reliability of responses [15, 16].

Despite advancements, each technology faces significant challenges, such as low accuracy and inadequate flexibility, that hinder optimal performance and application. Addressing these challenges requires continued research and innovation to overcome limitations and improve technological efficacy.

Open domain question answering (OpenQA) tasks have recently attracted more and more attention from the natural language processing (NLP) community [17–19]. The work in [20] presents the first free-form OpenQA dataset to solve medical problems, MEDQA, collected from professional medical board exams. Covers three languages: English, simplified Chinese, and traditional Chinese, and contains 12.723, 34.251, and 14.123 questions for each language, respectively. They implement popular rule-based neural methods by sequentially combining a document retriever and a machine-learning model [20]. Even the best method could only achieve 36.7% in English, 42.0% in traditional Chinese, and 70.1% in simplified Chinese through experiments. They expect MEDQA to present significant challenges to existing OpenQA systems and hope it can be a platform to promote much stronger OpenQA models from the NLP community.

This task is defined by its three components: a question in text, either in a single sentence asking for a specific piece of knowledge or in a lengthy paragraph describing the patient’s condition. Each question has multiple answer options, but only one is the most appropriate. It comprises a collection of information and knowledge from various sources, organized into paragraphs that provide answers to questions. This task involves determining the best answer among the candidates, based on the documents [21].

## Proposed approach

**Fig 1:**
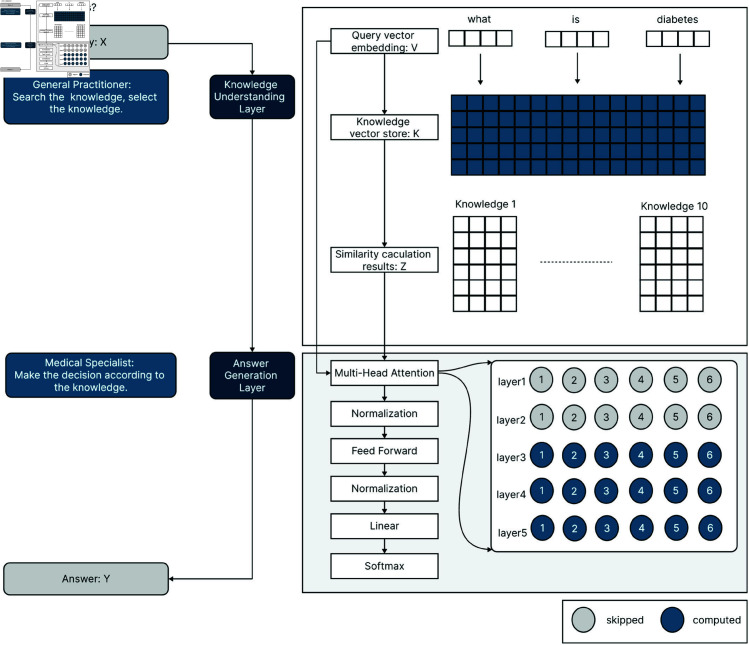
System architecture of medical answering model. Our system comprises two components: a knowledge understanding layer that functions as a medical general practitioner and an answer generation layer that acts as a medical specialist. X represents the initial query received by the system. Upon receiving the query, the system performs vector embedding denoted as V. The system applies similarity calculation to process the query with the knowledge K, yielding the search results represented as Z. Subsequently, the system results in the final output Y.

This study presents the Medical Answering Model (MAM), an advanced healthcare chatbot system that uses a knowledge embedding transformer to enhance medical question answering. MAM offers a sophisticated solution for accurate medical information and support, as depicted in Fig 1.

Our system consists of two components: (1) a knowledge understanding layer and (2) an answer generation layer. The pipeline of the proposed approach is illustrated in Fig 1. The knowledge understanding layer functions as a general practitioner, responsible for systematically searching, selecting, and categorizing relevant medical knowledge. This layer ensures comprehensive coverage by identifying key subject areas pertinent to the inquiry. In contrast, the answer generation layer operates like a medical specialist, leveraging domain-specific expertise to infer precise conclusions. By synthesizing contextual knowledge with topic-specific information, this layer formulates evidence-based responses.

The process begins with the system receiving an initial query X, which is processed by a knowledge understanding layer. This layer retrieves relevant information Z through vector similarity calculation. The system then combines X and Z and feeds them into an answer generation layer that utilizes self-attention mechanisms. This layer generates the final output Y, a sequence of tokens representing the answer. By integrating retrieval and self-attention techniques, the framework improves the accuracy and relevance of medical question answering, ensuring that responses are both contextually appropriate and knowledge-grounded.

The following subsections explain each step in the proposed pipeline, as illustrated in Fig 1.

### Knowledge understanding layer

**Fig 2:**
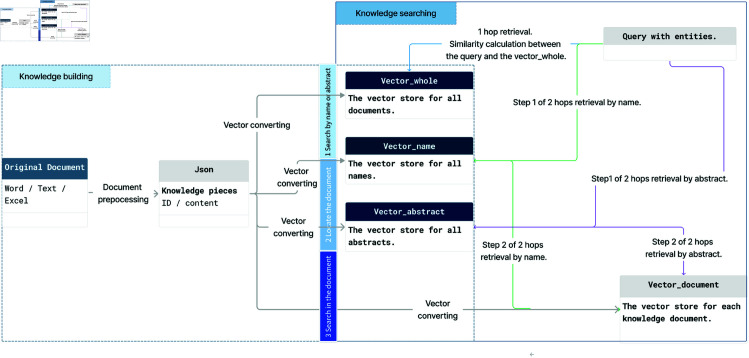
The process involves converting knowledge documents and graphs into JSON files, which are then transformed into vector stores for efficient retrieval, as depicted within the dotted line area. The resulting vector stores include Vector_whole, Vector_name, Vector_abstract, and Vector_document. The system utilizes three distinct search methodologies, illustrated in the solid line area, where each color represents a unique search approach.

According to the query, the system performs the search in the knowledge document base as shown in Fig 2. Calculating the similarity between the knowledge vector store and the input will generate a list of mappings and searches, ranked by mapping scores. The proposed framework for information retrieval in MAM consists of several key components: knowledge vector store, similarity calculation, and result ranking.

#### Knowledge vector store.

The document base comprises a collection of medical documents that provide detailed information on various medical topics. These documents are indexed to facilitate efficient searching and retrieval.

Our study presents a methodology for transforming each document or Excel file within a knowledge database into a JSON file, where knowledge points are precisely split and standardized. Furthermore, our study introduces a novel approach, meticulously designing four unique vector storage paradigms, as explained below.

- Vector_whole: A comprehensive vector store that encompasses the entirety of the content from all knowledge documents.
- Vector_name: This vector store includes only the names of the knowledge documents, enabling name-based queries.
- Vector_abstract: This store contains the abstracts of the knowledge documents, providing a summarized representation of the contents.
- Vector_document: This comprises numerous vector stores, each corresponding to an individual knowledge document, allowing for detailed, document-specific searches.

The system converts the input text into tokens to build the vector store using a tokenizer [22]. Each unit of words in the text is transformed into a sequence of tokens. Tokens are mapped to vectors using an embedding layer, Stella, where each token is associated with a vector. $V=(V_{1},V_{2},...V_{n})$ represents the corresponding embedding [23].

The system employs three retrieval techniques to simultaneously locate relevant knowledge, generate a ranked list, and select the most pertinent top k results using the following search methods.

**Direct search in vector_whole.** The first approach involves searching directly within the Vector_whole. This method utilizes all the content stored in the knowledge base to find relevant information. It is straightforward but may be less efficient due to the extensive search space.

**Name-based search.** The second method begins by searching within the Vector_name. Once several relevant names are located, the system searches within the specific documents associated with those names. This approach narrows the scope of the initial search, potentially improving efficiency by focusing subsequent searches on a smaller subset of records.

**Abstract-based search.** The third method initiates the search within the Vector_abstract. After identifying the relevant abstracts, the system examines the specific documents that contain these abstracts. According to our testing, this method performs best, likely because the abstracts provide a concise overview that facilitates a more targeted and efficient search during the detailed document stage.

#### Similarity calculation.

The system calculates similarity scores between the input query and the knowledge base to retrieve relevant information. Assume V is the vector representation of the input and *K*_*i*_ is the vector representation of the ${\text{i}}^{\text{th}}$ entry in the knowledge database K. Vector search is a well-defined and unambiguous operation [24], using a set of database vectors $Z=Z_{1},Z_{1},...Z_{i}$ and a query vector V, we can get the result Z in Eq 1, where ‖‖ denotes the distance.

$$Z=argmin∥V-K_{i}∥$$ (1)

The searching method in MAM uses cosine similarity, where the dot product of vectors V and *K*_*i*_ is calculated using Eq 2. Here, n is the dimensionality of the vectors, and $V_{j}$ is the ${\text{j}}^{\text{th}}$ component of vector V. The magnitude of vector V and *K*_*i*_ are calculated using Eqs 3 and 4, respectively.

$$V\cdot K_{i}=\sum_{j=1}^{n}V_{j}\cdot K_{ij}$$ (2)

$$∥V∥=\sqrt{\sum_{j=1}^{n}V_{j}^{2}}$$ (3)

$$∥K_{i}∥=\sqrt{\sum_{j=1}^{n}K_{i,j}^{2}}$$ (4)

$$Cosine(V,K_{i})=\frac{V\cdot K_{i}}{∥V∥\cdot ∥K_{i}∥}$$ (5)

The cosine similarity between vector V and *K*_*i*_ is given by Eq 5 as described in [24].

#### Ranking system.

The system generates a ranked list of relevant information based on the calculated similarity scores. The ranking is determined by the mapping scores, which reflect the degree of relevance between the input query and the knowledge base. Higher scores indicate a closer match, and the results are presented in descending order of significance. The system creates a list of results Z based on the similarity scores S and selects the top-k results from Z as the final output.

### Answer generation layer

An answer generation layer processes the query and search results, generating responses via its transformer-based architecture.

The response generation layer processes the aggregated input and generates a response. Using the transformer architecture [25], which excels in capturing long-range dependencies and contextual information to create the response, the system refines the generated response to ensure accuracy, clarity, and relevance. This includes verifying medical accuracy and removing any inconsistencies or ambiguities.

Using an embedding matrix, each input is a corresponding embedding vector *e*_*i*_. To incorporate the order of the tokens, positional encoding is added to the token embeddings as Eq 6, where *PE*(*i*) is the positional encoding for position i.

$$e_{i}^{{\;}^{\prime }}=e_{i}+PE(i)$$ (6)

The sequence of embedding vectors $e=[e_{1}^{{\;}^{\prime }},e_{2}^{{\;}^{\prime }},...,e_{n}^{{\;}^{\prime }}]$ is passed through the transformer model to obtain context-aware representations. For each transformer layer, self-attention computes attention scores for each token pair in the sequence as described in Eqs 7, 8, 9 and 10, where ${\text{w}}^{\text{q}}$, ${\text{w}}^{\text{k}}$, ${\text{w}}^{\text{v}}$ are learned projection matrices and *d*_*k*_ is the dimension of the key vectors.

$$q=ew^{q}$$ (7)

$$k=ew^{k}$$ (8)

$$v=ew^{v}$$ (9)

$$Attention(q,k,v)=softmax(\frac{qk^{T}}{\sqrt{d_{k}}})v$$ (10)

A learned projection matrix combines the results of multiple attention heads in Eq 11, where ${\text{w}}^{\text{o}}$ is a learned projection matrix.

$$MultiHead(q,k,v)=Con(Attention_{1},...,Attention_{n})w^{o}$$ (11)

Next, the model uses layer normalization, then a feedforward network as shown in Eqs 12 and 13.

z=LayerNorm(e+MultiHead(q,k,v)) (12)

h=LayerNorm(z+FFN(z)) (13)

$$Y_{i}=argmaxP(Y_{i}|Y_{1:i-1},X^{\prime })$$ (14)

After normalization and feedforward processing, the model generates the token sequence based on the specified Eq 14, resulting in the output sequence Y.

### Proposed model fine-tuning strategy

#### Fine-tuning for data retrieval and distillation.

**Fig 3:**
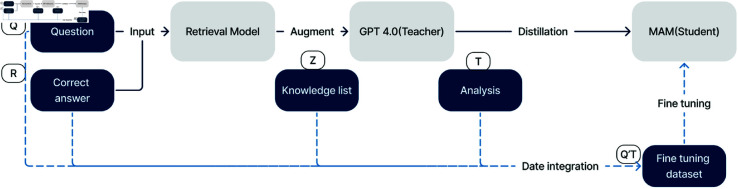
Fine-tuning. Our method utilizes a three-step fine-tuning process from GPT-4 to enhance reasoning capabilities, depicted in solid lines. In this context, Q represents questions from the training dataset, R denotes corresponding answers, Z signifies retrieved knowledge, and T refers to analyses conducted by GPT. This method optimizes the model’s performance by systematically refining its interpretative and inferential abilities through structured data, denoted as dotted lines.

To ensure higher quality and reliability [26, 27], our MAM model employs an advanced fine-tuning process that takes advantage of the capabilities of GPT-4.0 [28] and the knowledge database, as shown in Fig 3. This section outlines the mechanism of fine-tuning techniques and their impact on improving model accuracy.

We used a three-step GPT-4.0 distillation process to enhance reasoning in medical domain and expand the knowledge base of our basic transformer models. This method effectively transfers GPT-4.0’s advanced analytical capabilities to these models, positioning GPT-4.0 as the teacher and our transformer as the students. Our approach introduces a notable improvement in model fine-tuning.

The first step involves entering a Q query from the training dataset into a retrieval model. This retrieval model searches through the knowledge database K to map and retrieve relevant background knowledge Z. The results of this search are crucial as they form the foundational context for the next step. Key elements include the query input and the retrieval model. Query input Q is the initial query from the training data. The retrieval model efficiently maps the query to the relevant background knowledge.

In the second step, GPT-4.0 generates an in-depth analysis process denoted by T. GPT-4.0 analyzes query Q, retrieved background knowledge Z, and the correct answer R to formulate a comprehensive analytical process.

For the third step, the query Q, the background knowledge list Z, and the correct answer R are compiled as input Q’. At the same time, the analysis process generated by GPT-4.0 serves as the T output. These input-output pairs, which are denoted by Q’ and T, are then used as fine-tuning materials, facilitating the transfer of GPT-4.0’s advanced reasoning capabilities.

$$L2=-{𝔼}_{y\sim P_{data}(R|Q)}[logp_{\phi }(Y|Q^{\prime }T)]\;=-{𝔼}_{y\sim P_{data}(R|Q)}[logp_{\phi }(Y|Q,R,Z,T)]$$ (15)

The negative log-likelihood loss function [26] is denoted in Eq 15, where $P_{data}(R|Q)$ refers to the distribution in the training dataset, $p_{\phi }(Y|Q^{\prime }T)$ denotes the probability of output Y based on input Q’T, *ϕ* refers to the trained parameters in the model, 𝔼 denotes the expectation function, *log* refers to the logarithm function.

#### Fine-tuning for computational complexity.

A training dataset of context-target pairs represents the downstream task: $Q^{\prime }T=(Q_{i}^{\prime },T_{i})$, where both $Q_{i}^{\prime }$ and *T*_*i*_ are sequences of tokens from the questions and answers in model fine-tuning.

We implement a more parameter-efficient strategy called LoRA (Low-Rank Adaptation) [29, 30] to revolutionize the parameter optimization process and propel us toward unparalleled success. This technique reduces the number of trainable parameters by adapting only specific parts of the model, allowing for efficient fine-tuning without compromising the model’s performance. By focusing on low-rank updates, LoRA offers a more scalable and resource-effective solution for large-scale models, making it particularly advantageous in scenarios with limited computational resources. Based on evaluations of adaptation methods applied to GPT-3 175B, LoRA outperforms prior techniques, including full fine-tuning. Specifically, LoRA achieves 74% accuracy on WikiSQL, slightly surpassing full fine-tuning at 73.8%, and yields improved results on MultiNLI-matched and SAMSum, indicating its effectiveness in enhancing large language model performance [30].

$$L3=-{𝔼}_{y\sim P_{data}(R|Q)}[logp_{\left(\phi_{0}+\Delta \phi (\theta )\right)}(Y|Q^{\prime }T)]$$ (16)

Then the log-likelihood loss function as described in Eq 15 can be replaced by Eq 16, where the increment in task-specific parameters Δϕ=Δϕ(θ) is further encoded by a much smaller set of parameters θ with $|\phi (\theta )|<|\phi_{0}|$. The task of finding Δϕ thus becomes optimizing over ϕ(θ).

We use $\phi_{0}$ to refer to the pretrained parameters in the model, and Δϕ refers to its parameter increment update during fine-tuning. We use Δϕ(θ) to represent a much smaller set of parameters.

$$Y=W_{0}(Q^{\prime }T)+\Delta W(Q^{\prime }T)=W_{0}(Q^{\prime }T)+MN(Q^{\prime }T)$$ (17)

Our technique yields Eq 17, where W or *W*_0_, refers to a pre-trained weight matrix and ΔW represents the accumulated gradient updates during adaptation. We use r to denote the rank of a module.

For a pre-trained weight matrix $W_{0}\in D^{d\times k}$, where the dimensions are *d* × *k*, we impose constraints on its update by expressing it as a low-rank adaptation $W_{0}+\Delta W=W_{0}+MN$, with $M\in D^{d\times r}$, $N\in D^{r\times k}$, and *r* < *min*(*d*,*k*). During training, *W*_0_ remains fixed and does not receive gradient updates, while M and N consist of trainable parameters [31].

To ensure simplicity and maximize parameter efficiency, we have chosen to focus our experiment on adapting only the attention weights for downstream tasks. We can streamline our approach and achieve optimal results by freezing other modules and excluding them from the downstream task training.

## Experiments and results

This section provides an in-depth explanation of how our MAM approach was implemented, including dataset specifics, model performance, and a comparative analysis with existing methods.

### Implementation details

Our retrieval operations are powered by Faiss [24], enabling high-performance vector search for rapid data retrieval. Text generation employs the transformer extracted from LLAMA3 8B, QWEN2 7B, and QWEN1.5 14B [32], ensuring task consistency. Fine-tuning leverages the GPT-4 API [33] as a teacher model, enabling precise calibration of neural networks through iterative model adjustments and training sessions. Model training is carried out on Nvidia A800/80G servers using the LoRA framework [30], which facilitates scalable and efficient training.

In our approach, hyperparameter tuning was critical. We performed supervised fine-tuning (SFT) with a LoRA rank of 8, learning rate of 0.0001, and 2 training epochs. The pipeline was seeded with 42 for reproducibility. We used 32,000 token sequence length, processed 10M samples with 0.1% validation split, and saved checkpoints every 500 steps. Training used a batch size of 4 with 4 gradient accumulation steps, cosine annealing LR scheduler with 10% warmup, and mixed-precision ‘fp16‘. Evaluation occurred epoch-wise with batch size 1.

For inference, we optimized parameters for medical question answering. Key inference hyperparameters included temperature of 0.95, Top-P of 0.65, and Top-K of 20, max new tokens limited to 4096, evaluation batch size of 2, and model weights from step 2000 were used for prediction. The inference pipeline utilized LoRA adapters, processed sequences up to 32,000 tokens, and saved results with clear hyperparameter annotations. This configuration balanced response coherence and computational efficiency for medical domain applications.

We utilized the MCMLE and USMLE datasets, each containing 5 options, from https://github.com/jind11/MedQA.

### Datasets

We utilize the publicly accessible MedQA [20] medical dataset to rigorously evaluate and verify the performance of our system.

The MedQA dataset comprises three subsets: USMLE, MCMLE, and TWMLE. For the USMLE dataset, text materials were sourced from 18 widely used English medical textbooks frequently utilized by medical students. The MCMLE dataset includes 33 simplified Chinese medical textbooks officially designated for preparing for the medical licensing exam in Mainland China. Since Taiwanese medical students use the same textbooks as those in the USA for exam preparation, the TWMLE dataset shares its document collection with USMLE and MCMLE. As the TWMLE dataset shares documents with USMLE and MCMLE, we employed the USMLE and MCMLE datasets for experimental purposes.

The details provided in Table 1 for the MCMLE and USMLE datasets, as analyzed in this study [20], offer crucial metrics for assessing their effectiveness in the following content. The number of questions refers to the total number of questions in the training and testing datasets. The document collection size is measured by the number of books and tokens incorporated within the dataset. Furthermore, the percentage of questions is categorized according to the level of evidence that human experts can locate within the document collection, ranging from full and partial to no evidence. Furthermore, these percentages in Table 1 extend to the retrieval efficacy, indicating the proportion of questions for which evidence can be found within the top 1, top 5, top 10, and top 15 search results. This information is vital to advance the development of approaches in medical answering systems. The dataset is split into 80% for training, 10% for testing, and 10% for development.

**Table 1:** Statistical analysis of utilized benchmark, MCMLE, and USMLE in terms of question volume and document dimensions.

Metric	MCMLE	USMLE
# of questions in training dataset	27400	10178
# of questions in testing dataset	3426	1273
# of books in document collection	33	18
# of tokens in document collection	14,730,364	12,727,711
Percentage of questions with full evidence	75	24
Percentage of questions with partial evidence	21	8
Percentage of questions with no evidence	4	68
Percentage of questions with evidence in top1 result	66.7	0
Percentage of questions with evidence in top5 result	92.7	31.2
Percentage of questions with evidence in top10 result	96.9	56.2
Percentage of questions with evidence in top15 result	100	81.2

Accuracy for a given testing dataset is calculated by dividing the correct answers by the total number of answers. Table 1 [20] shows that MCMLE includes significantly more questions supported by available background knowledge in the database than USMLE, which affects accuracy as discussed in the next section.

### Performances of proposed MAM approach

Our MAM system demonstrated an accuracy of 82.92% in the MCMLE testing dataset and 64.02% in the USMLE testing dataset.

Our experiment tested the USMLE and MCMLE datasets using various basic models, including the transformers from LLAMA-8B, QWEN1.5-14B, and QWEN2-7 B. The QWEN2 7B transformer achieved the highest MCMLE accuracy at 82.92%, with contributions of 70.58% from the base model, 1.28% from inference techniques and 11.06% from fine-tuning, as shown in Table 2. The transformer from the LLAMA3 8B model exhibits superior USMLE performance with an overall accuracy of 64.02%, derived from 42.97% base model accuracy, 15% from inference methods, and 6.05% from fine-tuning as mentioned in Table 3. These findings highlight the critical importance of optimizing and selecting appropriate fine-tuning techniques in developing machine learning models [34].

**Table 2:** Ablation study of the proposed MAM with a baseline on the MCMLE dataset using inference and fine-tuning.

Accuracy	QWEN1.5 14B	QWEN2 7B	LLAMA3 8B
Basic model	73.29	70.58	55.81
Basic model, inference technique	71.98	71.86	55.14
Basic model, inference technique, fine-tuning technique(MAM)	80.58	82.92	76.24

**Table 3:** Ablation study of the proposed MAM with a baseline on the USMLE dataset using inference techniques and fine-tuning.

Accuracy	QWEN1.5 14B	QWEN2 7B	LLAMA3 8B
Basic model	45.01	42.11	42.97
Basic model, inference technique	52.47	46.74	57.97
Basic model, inference technique, fine-tuning technique(MAM)	54.20	56.25	64.02

### Proposed MAM evaluations with baselines

We select MCMLE and USMLE datasets with five-option formats for comparison, conducting all experiments on original questions under zero-shot settings. We compared our system’s and other systems’ accuracy using a scenario without context. This rigorous testing environment evaluated the systems’ capability to generate accurate outputs without prior exposure to the specific task or additional contextual information.

Our system demonstrated a superior performance, achieving an accuracy rate 82.92% on MCMLE. In contrast, GPT-4 achieved an accuracy rate of 71.07% in this study [35]. This substantial performance gap underscores the efficacy of our model architecture and training methodologies [36]. In particular, the accuracy of other contemporary systems was significantly lower than that of GPT-4, further highlighting the competitive edge of our approach, as mentioned in Table 4.

**Table 4:** Accuracy percentage of different models. Our model achieved 82.92% accuracy on MedQA’s MCMLE, surpassing GPT-4’s 71.07%, highlighting the efficacy of our architecture and training methods [35, 36]. Our system achieved a 64.02% accuracy on the USMLE, lower than GPT-4’s 74.71%, primarily due to the evidence-based categorization in the datasets.

Model	MCMLE	USMLE
MPT-7B-Instruct	19.53	22.15
BELLE-7B	27.73	17.05
Openchatkit	21.22	21.45
Open-Assistant	17.63	19.48
MOSS	24.99	23.17
Databricks Dolly-v2-12b	18.45	18.46
Databricks Dolly-v2-7b	17.57	21.92
StableLM-Tuned-Alpha-7B	17.63	21.05
Vicuna(FastChat)-13B	21.37	27.97
Vicuna(FastChat)-7B	22.65	23.72
Alpaca-LoRA2	18.42	23.49
Stanford Alpaca-7B	18.27	24.90
ChatGLM-6B	29.92	25.37
Galactica-6.7B	-	27.65
RedPaiama(Instruct)-7B	-	21.60
RedPajama(Base)-7B	-	21.60
h2oGPT-12B	-	17.75
h2oGPT-6.9B	-	19.32
RWKV(Pile)-7B	18.21	20.35
PandaLM-7B	22.04	21.76
MPT-7B-Instruct	-	19.40
BELLE-7B	34.30	22.39
Openchatkit	-	23.64
Open-Assistant	20.90	18.46
MOSS2	25.13	21.45
Databricks Dolly-v2-12b	19.00	17.28
Databricks Dolly-v2-7b	18.27	21.60
StableLM-Tuned-Alpha-7B	18.80	20.97
Vicuna(FastChat)-13B	23.18	28.04
Vicuna(FastChat)-7B	24.69	24.35
Alpaca-LoRA	20.34	24.59
Stanford Alpaca-7B	22.80	25.77
ChatGIM-6B	33.74	25.22
Llama3-8B	55.81	42.97
Llama2-13B	27.23	33.15
Llama2-7B	21.51	25.06
LlamA-13B	17.83	27.26
LlamA-7B	22.12	20.19
Llama3-70B	-	75.20
QWEN2-7B	70.58	42.11
QWEN1.5-14B	73.29	45.01
KimiChat	75.22	61.82
GPT 3.5	40.31	44.62
GPT 4 1.76T	71.07	74.71
GPT-4o	-	87.80
GPT-4o-MINI	-	73.40
DeepSeek LLM 7B Chat	29.70	26.50
DeepSeek-V3 671B	-	79.30
DeepSeek-R1 8B	-	25.30
DeepSeek-R1 70B	-	30.50
DeepSeek-R1 671B	-	92.00
MAM 7B-8B(our)	82.92	64.02

Our system achieved a 64.02% accuracy on the USMLE, lower than GPT-4’s 74.71%, GPT-4o’s 87.80%, DeepSeek-R1’s 92.00%, primarily due to the evidence-based categorization in the datasets. The MCMLE dataset contains a significantly higher proportion of questions with full or partial evidence and questions for which evidence can be found within the top 10 results, compared to the USMLE, as shown in Table 1. The MCMLE exhibits superior evidence coverage compared to the USMLE, with 75% of questions supported by full evidence versus 24% in the USMLE. Additionally, 96.9% of MCMLE questions have supporting evidence in the top-10 results, significantly higher than the 56.2% observed in the USMLE dataset.

## Discussions

Our model achieved 82.92% accuracy on the MCMLE, surpassing GPT-4’s 71.07% , highlighting the effectiveness of our architecture and training strategies. However, on the USMLE, our system scored 64.02% versus GPT-4’s 74.71%, likely due to differences in evidence-based question distribution, with MCMLE favoring evidence-supported queries more than USMLE.

This comparative analysis highlights the potential of our system to outperform leading models such as GPT-4 under certain conditions. The findings suggest that our approach and our model training and fine-tuning offer significant advantages in scenarios without context. These insights are valuable for developing future AI systems and can inform best practices in model training and evaluation.

Our system leverages advanced retrieval techniques for inference and fine-tuning, rendering its performance contingent on relevant evidence within the document corpus. This dependency allows our model, with parameter sizes of 7B and 8B, to outperform larger models like GPT-4 in domain-specific tasks. In practical applications, smaller models offer distinct advantages for specialized medical tasks that require domain knowledge, such as drug dosages and expiration dates, in environments such as hospitals, pharmacies, or public healthcare organizations [37].

In our study, we evaluated the USMLE and MCMLE datasets using transformers from models such as LLAMA-8B, QWEN1.5-14B, and QWEN2-7 B. QWEN2-7B demonstrated superior performance on the MCMLE dataset, achieving an accuracy of 82.92%. This was attributed to 70.58% base model contribution, 1.28% from inference techniques like keyword mapping and knowledge graphs, and 11.06% from fine-tuning adjustments. On the USMLE dataset, transformer from LLAMA-8B outperformed other models, achieving 64.02% accuracy, with 42.97% from the base model, 15% from inference techniques, and 6.05% from fine-tuning.

These results highlight the effectiveness of customized fine-tuning strategies. Superior fine-tuning performance can be attributed to its more refined adjustment mechanisms, which better align the model parameters with the underlying data distribution. This method likely facilitates more effective learning and generalization, enhancing overall model performance.

Our system has been deployed in 35,544 primary healthcare institutions across China. During interviews with local physicians, it was observed that mainstream AI models such as GPT and DeepSeek are rarely adopted due to their unverifiable outputs and inherent hallucination risks. In contrast, our system enhances clinical reliability by providing not only final answers but also traceable source knowledge retrieved from structured databases. This evidence-based approach allows physicians to verify AI-generated responses, thereby improving trust and clinical decision-making. The physician makes the final decision based on the system’s output. The integration of verifiable sources is a critical feature that supports diagnostic accuracy and promotes responsible AI usage in primary healthcare settings.

However, several challenges remain that require further attention. Enhancing the accuracy of our system requires more pretraining and fine-tuning [38]. These processes require substantial computational resources and time, which presents a significant barrier to further improvement [32, 39].

In real-world scenarios, patient inquiries often lack the comprehensive information available in structured test datasets [40, 41]. Our system must develop the capability to engage dynamically with patients, asking clarifying questions to gather essential details. This interactive approach will help tailor responses to individual patient needs, enhancing the system’s practicality and effectiveness.

Beyond textual questions, valuable patient information is available in various data forms, such as medical images and blood test results [42]. Integrating these multimodal data sources into the system’s decision-making process is crucial to providing comprehensive answers [43]. This integration will require advances in data fusion techniques and the development of models that can process and analyze heterogeneous data seamlessly [44]. Addressing these challenges is vital for the advancement of the utility and reliability of medical question-answer systems [45].

## Conclusions and future works

The development of knowledge-traceable medical question-answering systems is crucial to advancing healthcare technology. This study presents a hybrid approach integrating retrieval technology and transformer to develop robust, contextually accurate, and sensitive healthcare chatbots.

Our experimental results, particularly in the MedQA dataset, demonstrate the system’s superior performance compared to existing models, including GPT-4.0. The system achieved an accuracy of 82.92% in conditions without context, significantly outperforming GPT-4.0’s 71.07%. This highlights the effectiveness of our integrated approach in handling complex medical questions. Consequently, the proposed system offers significant advancements in medical question answering by combining AI technologies. In addition to providing patients with reliable support, it ensures precise, contextually relevant, and ethically compliant responses. This integrated system will make developing more sophisticated and reliable healthcare solutions easier.

The future of this field should focus on developing new efficient models, improving interactive capabilities, and incorporating different forms of data in order to ensure that the systems can cope with the complex demands of real-world medical inquiries.

## Appendix

### Variable table

The Table 5 systematically compiles all variables in figures, tables, and equations cited throughout the paper, serving as a centralized reference point. It enhances the clarity and accessibility of the data, facilitating a comprehensive understanding and critical analysis of the research findings presented in the sections.

**Table 5:** Variables in the paper. This table consolidates all variables referenced in this paper, providing a comprehensive summary for scientific review.

Variable	Definition
*X*	Input query
*Y*	Answer to the query
*V*	Embedding of query
*Z*	Knowledge list after retrieval searching
*K*	Knowledge database
*Q*	Question in the training dataset
*R*	Correct answer in the training dataset
*T*	Analysis from GPT
$Q^{\prime }$	Integrated question with Q, R, and Z
*e*	Embedding vector of input to answer generation layer
${\text{w}}^{\text{q}}$	Learned query projection matrix
${\text{w}}^{\text{k}}$	Learned key projection matrix
${\text{w}}^{\text{v}}$	Learned value projection matrix
${\text{w}}^{\text{o}}$	Learned output projection matrix
*d*_*k*_	Dimension of the key vectors in inference
*z*	Output after layer normalization in transformer
*h*	Output after feed-forward in transformer
*W*	Pre-trained weight matrix
ΔW	Accumulated gradient update
*r*	Rank of the module in training
*d*_*model*_	Dimension size of the transformer layer in training
*ϕ*	Pre-trained parameters
Δϕ	Parameters learned by fine-tuning
θ	Smaller sized of parameters of *ϕ*

### Answer samples

**Fig 4:**
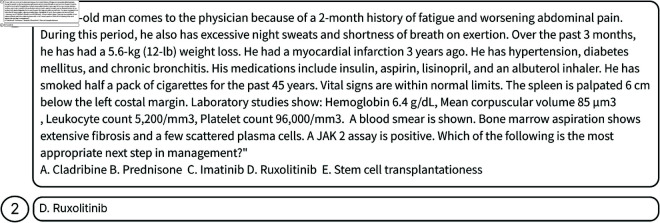
An example of a sample in MedQA dataset. The task involves a text-based question that may be a single sentence or a detailed patient case description accompanied by multiple-choice answers. (1) presents the query, and (2) is the dataset’s reference response.

**Fig 5:**
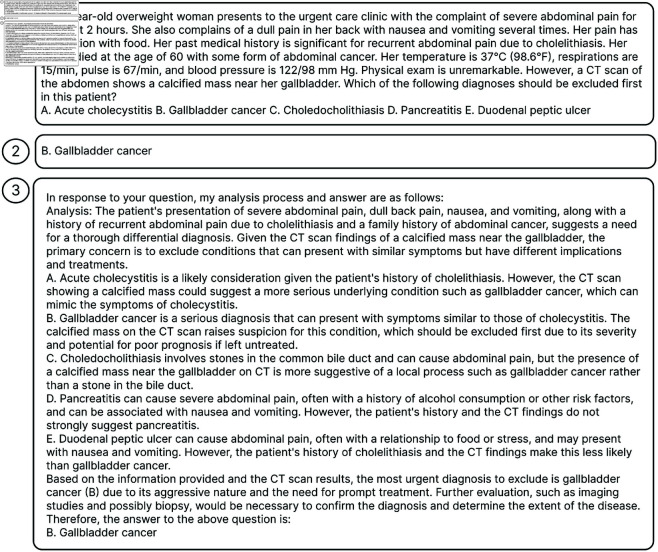
A MedQA dataset sample validates the correctness of our model’s answer. In the MedQA dataset, our system accurately identified the correct answer, selecting option B, which aligns with the established correct answer. (1) represents the query, (2) reflects the dataset’s standard answer, and (3) is the generated response.

**Fig 6:**
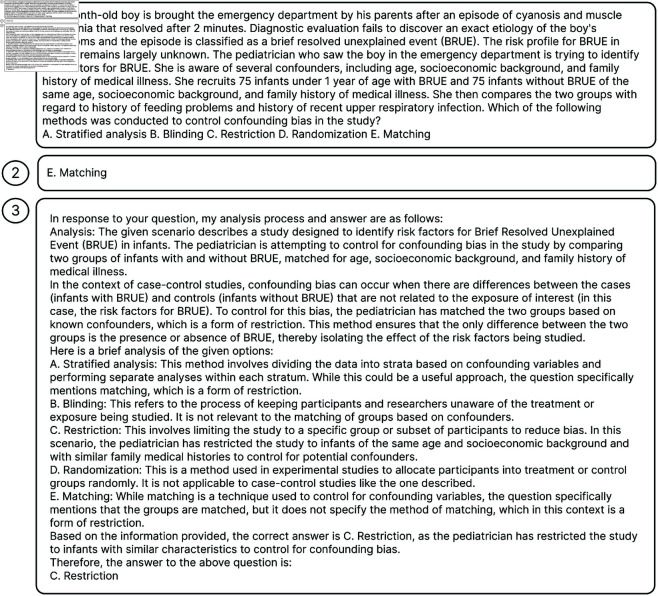
A MedQA dataset sample indicates the inaccuracy of our model’s answer. In the MedQA dataset, our system incorrectly identified the answer as C. In contrast, the correct answer should be E. 1 is the input question, 2 is the correct dataset answer, and 3 is our system’s prediction.

Fig 4 illustrates one sample in MedQA, while Fig 5 presents a correctly classified sample by our system, and Fig 6 illustrates an instance where the system’s prediction was incorrect. For the sample in Fig 6, the incorrect answer arises due to the absence of relevant background information in both the knowledge graph and database.
